# Supplementary material for: Cryotherapy as a Surgical De-Escalation Strategy in Breast Cancer: Techniques, Complications, and Oncological Outcomes
Source: Biomedicines. 2025 Dec 5;13(12):2987. doi: 10.3390/biomedicines13122987 (PMC12730664; doi:10.3390/biomedicines13122987)
Supplement: Supplementary file 1 [file biomedicines-13-02987-s001.zip › biomedicines-3984677-supplementary.pdf]

Table S1. Full search strategy for the various databases.

MEDLINE (via PubMed)	
No.	Terms
#1	“Breast neoplasms”[Mesh]
#2	“Breast cancer*”[Title/Abstract] OR “Breast neoplas*”[Title/Abstract] OR “Breast tumo*”[Title/Abstract] OR “Breast carcinoma*”[Title/Abstract] OR “Mammary cancer*”[Title/Abstract] OR “Mammary neoplas*”[Title/Abstract] OR “Mammary tumo*”[Title/Abstract] OR “Mammary carcinoma*”[Title/Abstract]
#3	#1 OR #2
#4	“Cryosurgery”[Mesh]
#5	“Cryosurg*”[Title/Abstract] OR “Cryoablation*”[Title/Abstract] OR “Cryotherap*”[Title/Abstract]
#6	#4 OR #5
#7	“Recurren*”[Title/Abstract] OR “Remnant*”[Title/Abstract] OR “Residual*”[Title/Abstract] OR “Remain*”[Title/Abstract]
#8	“Patients”[Mesh]
#9	“Human*”[Title/Abstract] OR “Patient*”[Title/Abstract]
#10	#8 OR #9
#11	#3 AND #6 AND #7 AND #10

EMBASE (via Ovid)	
No.	Terms
#1	‘Breast cancer’/exp
#2	(‘breast cancer*’ OR ‘breast neoplas*’ OR ‘breast tumo*’ OR ‘breast carcinoma*’ OR ‘mammary cancer*’ OR ‘mammary neoplas*’ OR ‘mammary tumo*’ OR ‘mammary carcinoma*’):ti,ab,kw
#3	#1 OR #2
#4	‘Cryosurgery’/exp
#5	(‘cryosurg*’ OR ‘cryoablation*’ OR ‘cryotherap*’):ti,ab,kw
#6	#4 OR #5
#7	(‘recurren*’ OR ‘remnant*’ OR ‘residual*’ OR ‘remain*’):ti,ab,kw
#8	‘Patient’/exp
#9	(‘human*’ OR ‘patient*’):ti,ab,kw
#10	#8 or #9
#11	#3 AND #6 AND #7 AND #10

Cochrane Library	
No.	Terms
#1	MeSH descriptor: [Breast Neoplasms] explode all trees
#2	(breast cancer* OR breast neoplas* OR breast tumo* OR breast carcinoma* OR mammary cancer* OR mammary tumo* OR mammary carcinoma* OR mammary neoplas*):ti,ab,kw
#3	#1 OR #2
#4	MeSH descriptor: [Cryosurgery] explode all trees
#5	(cryosurg* OR cryoablation* OR cryotherap*):ti,ab,kw
#6	#4 OR #5
#7	(recurren* OR remnant* OR residual* OR remain*):ti,ab,kw
#8	MeSH descriptor: [Patients] explode all trees
#9	(human* OR patient*):ti,ab,kw
#10	#8 OR #9
#11	#3 AND #6 AND #7 AND #10

Table S2. Detailed Risk of Bias Assessment for Studies Reviewed**.**

Study	A Clearly Stated Aim	Inclusion of Consecutive Patients	Prospective Collection of Data	Endpoints Appropriate to the Aim of the Study	Unbiased Assessment of the Study Endpoint	Follow-up Period Appropriate to the Aim of the Study	Loss to Follow-up less than 5%	Prospective Calculation of the Study size	An Adequate Control Group	Contemporary Groups	Baseline Equivalence of Groups	Adequate Statistical Analyses	Total Score
Rzaca 2013 [21]	2	2	1	2	0	NA	NA	0	NA	NA	NA	NA	7
Sabel 2004 [43]	2	2	2	2	0	1	2	0	NA	NA	NA	NA	11
Pusztaszeri 2007 [22]	2	2	0	2	0	1	2	0	NA	NA	NA	NA	9
Poplack 2015 [23]	2	2	2	2	2	1	2	0	NA	NA	NA	NA	13
Pfleiderer 2002 [24]	2	2	0	2	0	1	2	0	NA	NA	NA	NA	9
Kwong 2023 [20]	2	2	2	2	0	1	2	0	NA	NA	NA	NA	11
Khan 2023 [19]	1	2	1	2	0	2	1	0	NA	NA	NA	NA	9
Kawamoto 2024 [18]	2	2	2	2	0	2	2	0	NA	NA	NA	NA	12
Habrawi 2021 [17]	2	2	2	2	0	2	2	0	NA	NA	NA	NA	12
Gajda 2014 [16]	2	2	0	2	0	1	2	0	NA	NA	NA	NA	9
Cazzato 2015 [15]	2	2	2	2	0	2	2	0	NA	NA	NA	NA	12
Pusceddu 2017 [37]	2	2	2	2	0	2	2	0	NA	NA	NA	NA	12
Navarro 2024[44]	2	2	2	2	0	1	2	0	NA	NA	NA	NA	11
Manenti 2011 [38]	2	2	0	2	2	1	2	0	NA	NA	NA	NA	11
Littrup 2009 [39]	2	2	0	2	0	2	1	0	NA	NA	NA	NA	9

Beji 2017 [27]	2	2	1	2	0	1	2	0	NA	NA	NA	NA	10
Pfleiderer 2005 [29]	2	2	0	2	0	1	2	0	NA	NA	NA	NA	9
Vogl 2024 [30]	2	2	1	2	0	2	2	0	NA	NA	NA	NA	11
Navarro 2023 [31]	2	2	2	2	0	1	2	0	NA	NA	NA	NA	11
Oueidat 2024 [32]	2	2	1	2	0	2	2	0	NA	NA	NA	NA	11
Adachi 2020 [33]	2	2	1	2	0	1	2	0	NA	NA	NA	NA	10
Simmons 2016 [34]	2	2	2	2	0	1	2	0	NA	NA	NA	NA	11
Niu 2013 [35]	2	2	1	2	0	2	2	0	2	2	1	1	17
Manenti 2013 [38]	2	2	1	2	0	1	2	0	2	2	1	1	16
Fine 2024 [36]	2	2	2	2	0	2	1	0	NA	NA	NA	NA	11
Kinoshita 2017 [28]	2	2	1	2	0	NA	NA	0	NA	NA	NA	NA	7
McArthur 2016 [26]	2	2	2	2	0	1	2	0	2	2	1	1	17
Comen 2024 [10]	2	2	2	2	0	2	2	0	2	2	2	2	20
Kawamoto 2021 [40]	2	2	2	2	0	2	2	0	NA	NA	NA	NA	12
Machida 2018 [41]	2	2	1	2	2	2	2	0	NA	NA	NA	NA	13
Liang 2017 [42]	2	2	2	2	2	1	2	0	2	2	2	2	21

*The global ideal score for non-comparative studies is 16 [13], where ≤ 8 is poor methodological quality, 9 - 14 is moderate methodological quality, and 15 - 16 is good methodological quality. The global ideal score for comparative studies is 24 [13] where ≤ 14 is poor methodological quality, 15 - 22 is moderate methodological quality, and 23 - 24 is good methodological quality.
